# Characterization of DNA aptamers generated against the soft-shelled turtle iridovirus with antiviral effects

**DOI:** 10.1186/s12917-015-0559-6

**Published:** 2015-09-30

**Authors:** Pengfei Li, Lingli Zhou, Yepin Yu, Min Yang, Songwei Ni, Shina Wei, Qiwei Qin

**Affiliations:** Key Laboratory of Tropical Marine Bio-resources and Ecology, South China Sea Institute of Oceanology, Chinese Academy of Sciences, 164 West Xingang Road, Guangzhou, 510301 China; University of Chinese Academy of Sciences, 19 Yuquan Road, Beijing, 100049 China

**Keywords:** Soft-shelled turtle iridovirus, Aptamer, Targeted antiviral therapy

## Abstract

**Background:**

Soft-shelled turtle iridovirus (STIV) causes severe systemic disease in farmed soft-shelled turtles (*Trionyx sinensis*). More efficient methods of controlling and detecting STIV infections are urgently needed.

**Methods:**

In this study, we generated eight single-stranded DNA (ssDNA) aptamers against STIV using systematic evolution of ligands by exponential enrichment (SELEX).

**Results:**

The aptamers formed representative stem-loop secondary structures. Electrophoretic mobility shift assays and fluorescent localization showed that the selected aptamers had high binding affinity for STIV. Aptamer QA-36 had the highest calculated binding affinity (*K*_*d*_) of 53.8 nM. Flow cytometry and fluorescence microscopy of cell-aptamer interactions demonstrated that QA-12 was able to recognize both STIV-infected cells and tissues with a high level of specificity. Moreover, the selected aptamers inhibited STIV infection *in vitro* and *in vivo*, with aptamer QA-36 demonstrating the greatest protective effect against STIV and inhibiting STIV infection in a dose-dependent manner.

**Discussion:**

We generated DNA aptamers that bound STIV with a high level of specificity, providing an alternative means for investigating STIV pathogenesis, drug development, and medical therapies for STIV infection.

**Conclusions:**

These DNA aptamers may thus be suitable antiviral candidates for the control of STIV infections.

## Background

The Chinese soft-shelled turtle (*Trionyx sinensis*) has high nutritional and economic values and is cultured commercially in China and other Asian countries, such as Japan, Vietnam and Korea [1]. Moreover, the turtle’s unique body plan makes it a useful model organism for developmental and biological research [2–5]. However, the rapid growth of turtle aquaculture has led to outbreaks of viral, bacterial, and parasitic diseases that threaten the industry’s future development. Among these outbreaks, soft-shelled turtle iridovirus (STIV) isolated from *T. sinensis* with ‘red neck disease’ has caused great economic losses [1], indicating an urgent need for effective diagnostic and therapeutic agents to control STIV infections in aquaculture systems.

Aptamers are synthetic nucleic acids (single-stranded DNA (ssDNA) or RNA) or protein ligands, selected using systematic evolution of ligands by exponential enrichment (SELEX) technology, which was first reported in 1990 and since been widely used in many applications [6, 7]. Aptamers have distinct three-dimensional structures and are characterized by complex structural features including stems, loops, hairpins, and pseudoknots [8, 9]. Their high specificity, low immunogenicity and lack of toxicity mean that aptamers have been used as specific probes in many areas, such as diagnostics, pathogen detection, and cancer research [10–14]. Current knowledge of the viral life cycle has identified important markers during its infectious process [15–19], relating to viral replication, assembly and release. These markers may serve as target molecules for aptamer selection. RNA aptamers with therapeutic potential for viral hemorrhagic septicemia virus and Hirame rhabdovirus in fish have been generated using purified virus particles as targets [20, 21]. We previously selected a panel of DNA aptamers against purified SGIV particles and demonstrated their inhibitory effects on viral infection *in vitro* and *in vivo* [22].

The aim of the present study was to generate and characterize a panel of ssDNA aptamers against intact STIV using a SELEX iterative method. We investigated the specificities of aptamer-binding to STIV and to STIV-infected cells *in vitro* and to STIV-infected tissues *in vivo*. The selected aptamers demonstrated good potential as molecular probes for the development of diagnostics and as drug-delivery vectors for controlling STIV infection. We also evaluated aptamer-mediated cytotoxicity and the effects of the aptamers on STIV infection in cultured cells *in vitro* and in Chinese soft-shelled turtles (*Trionyx sinensis*), *in vivo*.

## Methods

### Ethics statement

All experimental procedures on turtles were approved by Ethical Committee of South China Sea Institute of Oceanology, Chinese Academy of Sciences. Procedures involving turtles were carried out in accordance with the guidelines issued by the Ethical Committee of Chinese Academy of Sciences, as described previously [22]. All sections of this report adheres to the ARRIVE guidelines for reporting animal research.

### Viruses and cell lines

STIV (strain 9701) and Singapore grouper iridovirus (SGIV, strain A3/12/98) were preserved in our laboratory [23, 24]. Fathead minnow (FHM) cells were grown and maintained in Leibovitz’s L-15 medium supplemented with 10 % fetal bovine serum (Gibco, Life Technologies, Carlsbad, CA, USA) at 28 °C, as described previously [25, 26]. Virus titer was determined based on the 50 % tissue culture infective dose (TCID_50_) [27].

### Initial library and primers for SELEX

The SELEX library (Sigma-Aldrich, St. Louis, MO, USA) comprised 44.7 nmol ssDNA, as described and used previously [22]. It included two primer-hybridization sequences and a central randomized 50-nucleotide sequence (N50) (5'-GACGCTTACTCAGGTGTGACTCG-N50-CGAAGGACGCAGAGAAGTCTC-3'). The 5′-primer (5′-GACGCTTACTCAGGTGTGACTCG-3′) was labeled with fluorescein isothiocyanate (FITC) or tetramethyl-6-carboxyrhodamine (TAMRA), and the 3'-primer (5'-GAGACTTCATCTGCGTCCTTCG-3′) was biotinylated.

### Whole-virus SELEX

The ssDNA aptamers were selected based on the SELEX protocol reported by Pan et al. (1995), with some modifications [28]. For the first selection cycle, ssDNA (10 nmol) was denatured at 94 °C for 10 min, cooled on ice for 10 min, and then dissolved in 200 μl binding buffer (100 mM NaCl, 2.5 mM MgCl_2_, 20 mM Tris–HCl (pH 7.5)). A polyvinylidene difluoride (PVDF) filter (0.1 μm)(EMD Millipore, Billerica, MA, USA) was pre-wetted and the dissolved ssDNA was passed through the filter to remove non-specific-bound ssDNA. The filtrate was collected and incubated with 100 μl purified STIV (10^9^ TCID_50_/ml) for 1 h on ice. Another pre-wetted filter was used to separate the unbound ssDNAs from the ssDNA-STIV complexes. After washing with binding buffer, the ssDNA-STIV complexes were collected from the filter with TN buffer (150 mM NaCl, 50 mM Tris–HCl (pH 7.5)), and heated at 95 °C for 5 min to dissociate the bound ssDNA. The dissociated aptamers were amplified by polymerase chain reaction (PCR) (25 cycles of denaturation at 94 °C for 1 min, annealment at 60 °C for 30 s, and extension at 72 °C for 1 min, followed by a final extension at 72 °C for 5 min). The sense ssDNA was then isolated by heating the PCR products at 95 °C for 5 min followed by immediate cooling on ice for 10 min. Pierce streptavidin magnetic beads (100 μl) (Takara-Bio, Shiga, Japan) were added to separate the biotin-conjugated antisense and sense ssDNAs using a MiniMACS Separator (Miltenyi Biotec, Cologne, Germany). Sense ssDNAs were collected and used for further selection. We enhanced the specificity and affinity of the selected aptamers by serially decreasing the incubation time and STIV and ssDNA contents.

### Selection and isolation of DNA aptamers

The final ssDNA pool was PCR-amplified using unlabeled primers. The PCR products were then ligated into the pMD18-T vector (Takara-Bio) and transformed into *Escherichia coli* (DH5α), as described previously [29]. A total of 200 isolated clones were picked and sequenced. DNA sequences present in more than two clones were chosen as candidate aptamers and synthesized by Life Technologies. The secondary structure of the aptamers was predicted using MFOLD software (http://mfold.rna.albany.edu/?q=mfold/DNA-Folding-Form), as described previously [22].

### Electrophoretic mobility shift assay

The specificity of aptamer-STIV binding was verified by electrophoretic mobility shift assay (EMSA), as described previously [20–22]. Each aptamer (7.5, 15 or 30 μg, respectively) was incubated with 100 μl purified STIV (10^9^ TCID_50_/ml) for 1 h on ice. The ssDNA-STIV complexes were then filtered through a 0.1-μm pre-wetted PVDF filter (EMD Millipore) to remove unbound aptamers. After elution from the filter with TN buffer, the aptamer-STIV complexes were loaded onto a non-denaturing gel (6 %) for polyacrylamide gel electrophoresis. The gel was stained with SYBR Green EMSA stain and visualized by UV epi-illumination at 312 nm, following the manufacturer’s instructions (Life Technologies). Aptamers incubated with SGIV (10^9^ TCID_50_/ml) served as controls to demonstrate aptamer-specific binding.

### Fluorescent localization of STIV-aptamer binding

The specificity of aptamer-STIV binding was further verified by fluorescent localization, as described previously [30], with some modifications. STIV was labeled with the aptamers as follows: FITC-aptamers were denatured at 95 °C for 5 min and cooled on ice for 10 min, followed by the addition of 100 μl purified STIV (10^9^ TCID_50_/ml) and incubation on ice for 1 h. The aptamer-STIV mixtures were passed through a pre-wetted 0.1-μm PVDF filter (EMD Millipore) and eluted from the filter by washing with TN buffer. The chemical dye Hoechst 33,342 was then added to label the virus. After washing three times, the samples (10 μl) were dropped onto a glass coverslip and imaged using a fluorescence microscope (Leica DMRXA, German) at an excitation wavelength of 488 nm (green for FITC) or 350 nm (blue for Hoechst 33,342). The FITC-library pool and SGIV served as controls.

### Measurement of STIV-biotinylated ssDNA aptamer interaction by enzyme-linked immunosorbent assay

The aptamer-STIV binding interaction was measured by enzyme-linked immunosorbent assay (ELISA), as described previously [31], with some modifications. 5′-Biotinylated ssDNA aptamers were synthesized by Life Technologies. The aptamers were heated at 95 °C for 5 min and cooled on ice for 10 min. STIV (10^9^ TCID_50_/ml) was incubated with each aptamer (200 nM) in binding buffer and the mixtures were passed through a pre-wetted 0.1-μm PVDF filter (EMD Millipore). After washing with binding buffer, the 5′-biotinylated aptamer-STIV complexes were eluted from the filter and transferred to 96-well plates (Pierce, USA). The bound aptamers were detected using streptavidin-conjugated horseradish peroxidase (HRP) (1:10,000, Pierce). After adding 50 μl 2 M H_2_SO_4_ to terminate the color reaction, the absorbance of each well was measured at 450 nm using an ELISA plate reader.

### Analysis of binding affinity

The binding affinities of the aptamers were analyzed as described previously [32]. 5′-Biotinylated aptamers at various concentrations (0–500 nM) were incubated with STIV (10^9^ TCID_50_/ml) for 40 min on ice. The biotinylated ssDNA-STIV complexes were then filtered through 0.1-μm pre-wetted PVDF filters (EMD Millipore) to remove unbound aptamers. After elution from the filter with TN buffer, the aptamer-STIV complexes were transferred to 96-well plates (Pierce) and detected using streptavidin-conjugated HRP (1:10,000, Pierce). The samples were incubated with TMB chromogen solution for 10 min. After adding 50 μl 2 M H_2_SO_4_ to terminate the color reaction, the absorbance of each well was measured at 450 nm using an ELISA plate reader. The library incubating with STIV were served as the control.. After subtracting the mean values of the control groups according to the equation: Y = BmaxX/(*K*_*d*_ + X), the apparent equilibrium dissociation constants (*K*_*d*_) of the aptamer-STIV interactions were calculated using SigmaPlot software [33]. Results for each aptamer was presented as the mean ± SD of three independent experiments.

### Cytotoxicity assays

Cytotoxicity analyses of the selected aptamers were based on the assays described by [34]. FHM cells in 96-well plates were incubated with each aptamer candidate at various concentrations (1–1000 nM) at 28 °C for 48 h, with untreated FHM cells as a control. To assess cell viability, 20 μl MTT solution (Takara-Bio) was added to each well and incubated for 4 h at 28 °C. The color change at 450 nm was measured using an ELISA plate reader. The results of at least three assays were averaged independently for each aptamer.

### Effect of aptamers on STIV infection in cultured cells

The inhibitory effects of the selected aptamers on STIV were assessed as described previously [22, 34]. Each selected aptamer (500 nM) was incubated with STIV (multiplicity of infection (MOI) = 0.5) and added simultaneously to FHM cells in a 24-well plate. SELEX library (500 nM) incubated with STIV (MOI = 0.5) or STIV alone (MOI = 0.5) were added to the cells as controls. The cells were examined by light microscopy at 48 h post-infection (p.i.), to detect the cytopathic effect (CPE). Mixtures of supernatant and cells in each well were collected to determine virus titers by TCID_50_ assay [28]. Data from three independent experiments were used to quantify the effects of each selected aptamer on virus infection.

### Protective effects of aptamers against STIV *in vivo*

Apparently healthy Chinese soft-shelled turtles (approximately 5 g) were obtained from a local aquaculture farm, Guangdong, China. The turtles were maintained in tanks with a closed re-circulating dechlorinated-water system and fed daily with a commercial diet. Chinese soft-shelled turtles were starved for 24 h prior to the experiment. Five groups of turtles (*n* = 30 each) were treated by intraperitoneal injection as follows: 60 μg selected aptamer incubated with 100 μl STIV (10^8^ TCID_50_/ml); 60 μg initial library incubated with 100 μl STIV (10^8^ TCID_50_/ml); 60 μg selected aptamer only; 100 μl STIV (10^8^ TCID_50_/ml) only; untreated controls. Each group was transferred to a separate aquarium with ample aeration and running water and mortality was recorded daily up to 10 days p.i. Turtles in each group were anaesthetized in ethyl 3-aminobenzoate methanesulfonate solution (60 mg/L). Liver and spleen tissues were harvested from each turtle, and washed with 10 ml phosphate-buffered saline (PBS). After being fixed with 10 % neutral buffered formalin, each tissue was processed as paraffin and frozen section, respectively, as described previously [22]. Liver and spleen tissues were also ground in phosphate-buffered saline (PBS) and then centrifuged, and the supernatant was collected and filtered through a 0.22-μm filter (Millipore). The filtrates were transferred to FHM cells at 28 °C and the CPE was examined daily.

### Flow cytometric analysis and fluorescent imaging

The specificity of aptamer candidate binding to STIV-infected cells was evaluated by flow cytometric analysis and fluorescence microscopy [34, 35]. The FITC-labeled aptamers were first denatured at 94 °C for 10 min and cooled on ice for 10 min. After washing three times with PBS (2 mmol/l KH_2_PO_4_, 10 mmol/l Na_2_HPO_4_ · 12H_2_O, 137 mmol/l NaCl, 1‰NaN_3_), STIV-infected FHM cells were incubated with FITC-labeled aptamers in 200 μl binding buffer on ice for 40 min, washed twice with PBS, and then resuspended in 400 μl PBS. FITC fluorescence was measured using a FACScan cytometer (BD Immunocytometry Systems, USA) by counting 20,000 events. The FITC-labeled initial library incubated with STIV-infected FHM cells were used as the control.

After denaturing at 94 °C for 10 min and cooling on ice for 10 min, FITC-labeled aptamers (300 nM) were incubated with 10^5^ STIV-infected FHM cells per well in 24-well plates at 4 °C for 1 h in the dark. The cells were then fixed with 4 % paraformaldehyde and washed three times with PBS. Fluorescent imaging of the cells was performed using a fluorescence microscope (Leica DMRXA, German) at an excitation wavelength of 488 nm (green for FITC) or visible light. The initial library incubated with STIV-infected FHM cells and aptamers incubated with normal FHM cells were used as the negative controls.

The protocols for TAMRA-labeled aptamers (300 nM) binding to STIV-infected tissues were similar to fluorescent imaging of the cells, at 560 nm excitation (red for TAMRA) and visible light. FITC-labeled initial library and normal tissues were used as controls.

### Statistical analysis

The experimental data are expressed as the mean ± standard deviation (SD). Differences between groups were compared by one-way analysis of variance. Values of *P* < 0.05 were considered to represent statistically significant differences. Statistical analysis was performed using SPSS, version 13.0 (IBM, Armonk, NY, USA).

## Results

### Selection of ssDNA aptamers against STIV

**Fig. 1:**
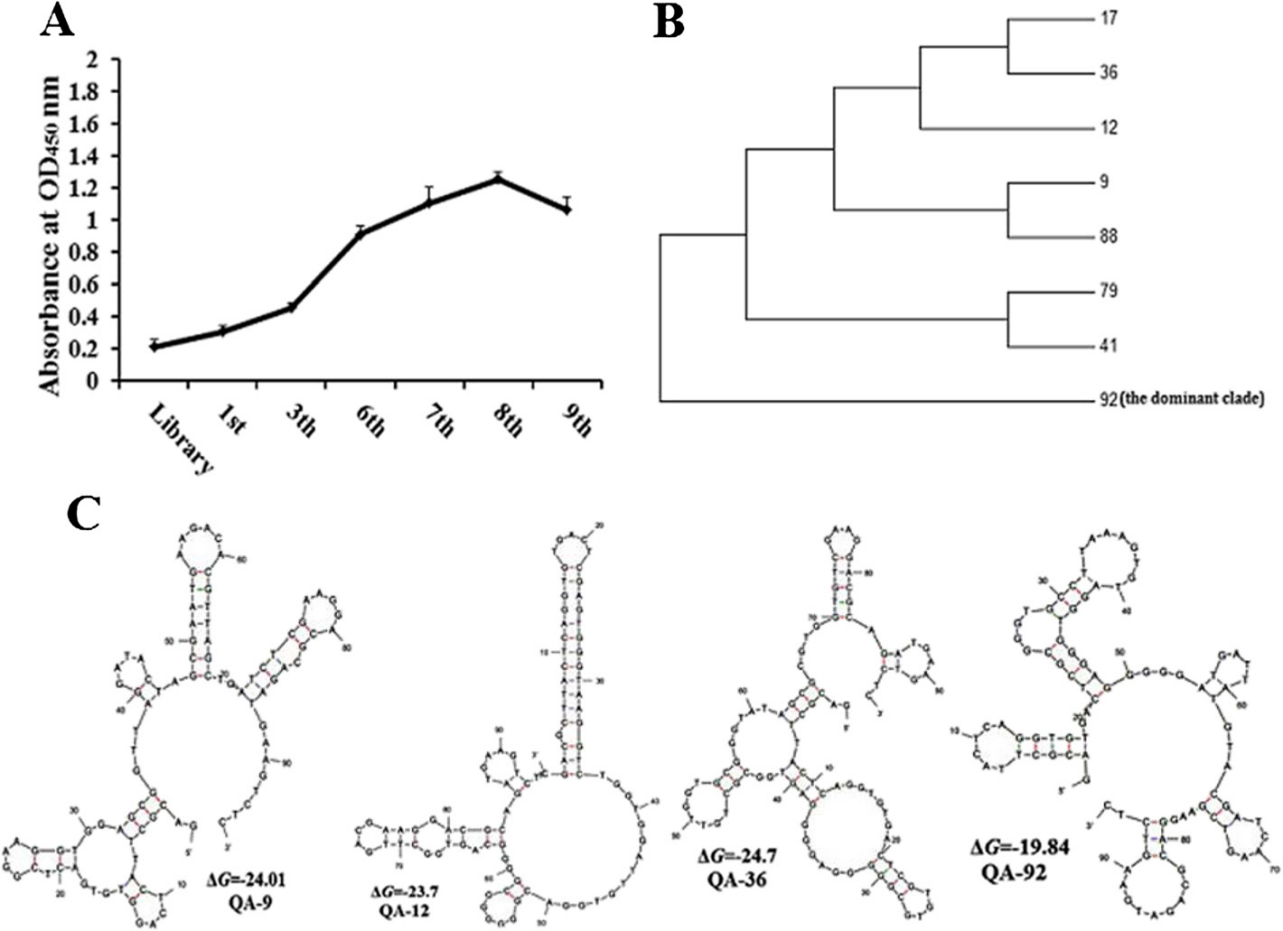
SELEX isolated eight aptamers that specifically targeted STIV particles. **a** Binding to STIV was enhanced in the higher selected pools, as demonstrated by ELISA. **b** Cluster analysis of the selected aptamers. **c** Secondary structures of aptamers QA-36, QA-92, QA-9, and QA-12. The stabilities of aptamer secondary structures were calculated as the free energy (Δ*G*)

Specific enrichment of each selected pool was monitored by ELISA. ssDNA binding increased as the selection cycles progressed, confirming that the selected pools were specific to STIV. ssDNA binding to STIV peaked at the 8th pool (Figure 1a), and eight ssDNA aptamers were isolated from the 8th pool (Table 1) based on their highly-specific STIV binding. We did an alignment of the selected aptamers, the consensus regions within the individual aptamers were also shown in Table 1, all aptamers held the specific motifs of “-GG-------------------------G----------T-”. The QA-92 aptamer comprised 26 % of the aptamer pool, while the other aptamers were less prevalent in the pool. Cluster analysis showed that QA-92 was a single clade that did not cluster with the other seven aptamers (Fig. 1b). QA-92, QA-9, QA-12 and QA-36 comprised 88 % of the aptamer pool and were selected for further analysis.

**Table 1:** Identification of ssDNA aptamers

Aptamer	Central randomized sequences	Frequency
QA-92	CG**GG**TGCCTTAAAGTGTAGGTGG**G**AGGGG**G**ATGA**T**TATGTACGATCAAGT	26 %
QA-9	GAAGGT**GG**AGCGGTTAGGATACTAGCGAATGAA**G**ACACGTTAGC**T**GATCT	23 %
QA-12	AGTG**GG**TAAGGTCTGGTGGATTGTGGACGGG**G**GGCGGGGCAG**T**GGCTTGA	21 %
QA-36	TGTGCG**GG**GGAGGGGAGTGGCGCTGTTGGTGCG**G**GTATAGCGCG**T**GGTGT	18 %
QA-17	AGAG**GG**TCGGTCGTAGTGGATTTGGCGCATT**G**TTCTGCGGGG**T**GGGAGGG	2 %
QA-79	GTC**GG**GACAGTGTTGGTCCTCAGGATCTCT**G**GGGCGCGGGG**T**TAAACAGT	2 %
QA-41	TGTTC**GG**GTTATTGCTCCTCCTTATT**G**TCACCT**G**GATG**T**ATGATCGTGTAG	4 %
QA-88	GGATAG**GG**GGCTCGCTCGTTCCGTAGC**G**ATGCAG**G**TTTC**T**TATTCACGAA	2 %
Consensus	-GG---------G-----T-	

Frequency indicates the percentage of the 8th selected pool comprised by each aptamer. Consensus regions within the representative aptamers are bold

Secondary structures and free energy (Δ*G*) values of the selected aptamers were calculated using the MFOLD program. The aptamers all formed representative stem-loop structures, and QA36 had the lowest Δ*G* value of −24.7 (Fig. 1c).

### Selected aptamers bound STIV with high specificity

**Fig. 2:**
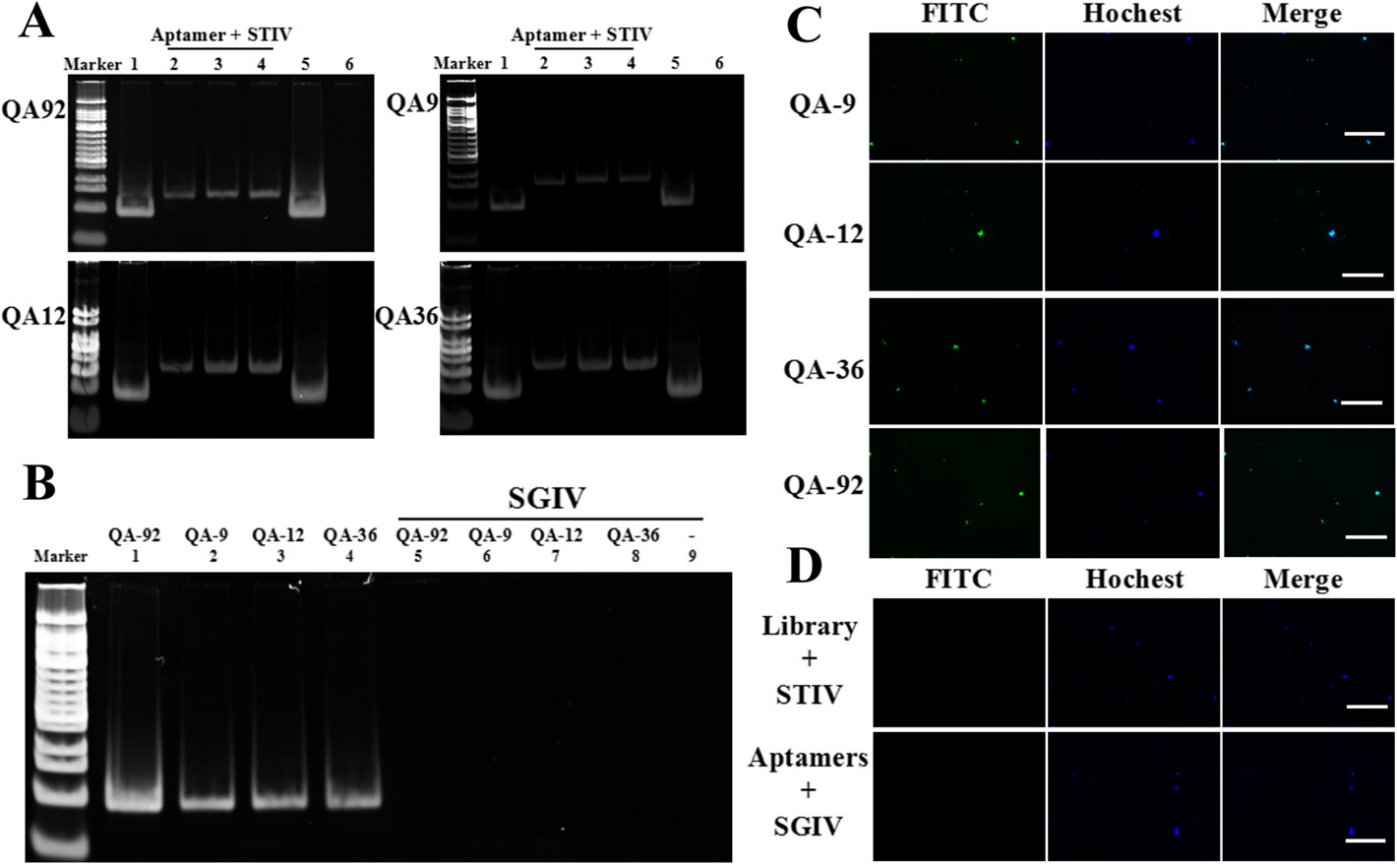
Analysis of binding specificity of anti-STIV ssDNA aptamers. **a** EMSA of selected aptamers incubated with STIV (lane 1, 7.5 μg aptamer only; lanes 2–4, STIV incubated with 7.5, 15 or 30 μg of each aptamer, respectively; lane 5, 30 μg of aptamer only; and lane 6, STIV only). **b** EMSA of selected aptamers incubated with SGIV. Note that no bands were visible in lanes 5–9, which proved the specificity of selected aptamers only recognizing STIV. **c**, **d** Fluorescent localization of STIV-aptamer binding in virus particles stained with Hoechst 33,342. **c** Imaging of FITC-aptamer-labeled STIV virus particles by fluorescence microscopy. **d** Controls of FITC-aptamers not binding to SGIV, and initial library not binding to STIV. (Bars = 50 μm)

Specific aptamer-STIV binding was demonstrated by EMSA assays. Free ssDNA aptamers appeared at the bottom of the gel (Fig. 2a, lanes 1 and 5). Only single bands were visible in the upper region of the gel in the case of aptamers incubated with STIV (Fig. 2a, lanes 2–4), indicating that the selected aptamers bound STIV. When the aptamers were incubated with a different virus (SGIV), no bands were observed in the gel, which proved the specificity of aptamers only recognizing STIV (Fig. 2b).

The specific binding of aptamers to STIV was also demonstrated by fluorescent imaging of FITC-aptamer-labeled STIV virus particles by fluorescence microscopy. STIV virus particles labeled with FITC-aptamers and Hoechst 33,342 were evident and colocalized (Fig. 2c). STIV virus particles incubated with FITC-library and SGIV virus particles incubated with FITC-aptamers as controls showed no colocalization (Fig. 2d).

### Binding affinities of selected ssDNA aptamers with STIV

**Fig. 3:**
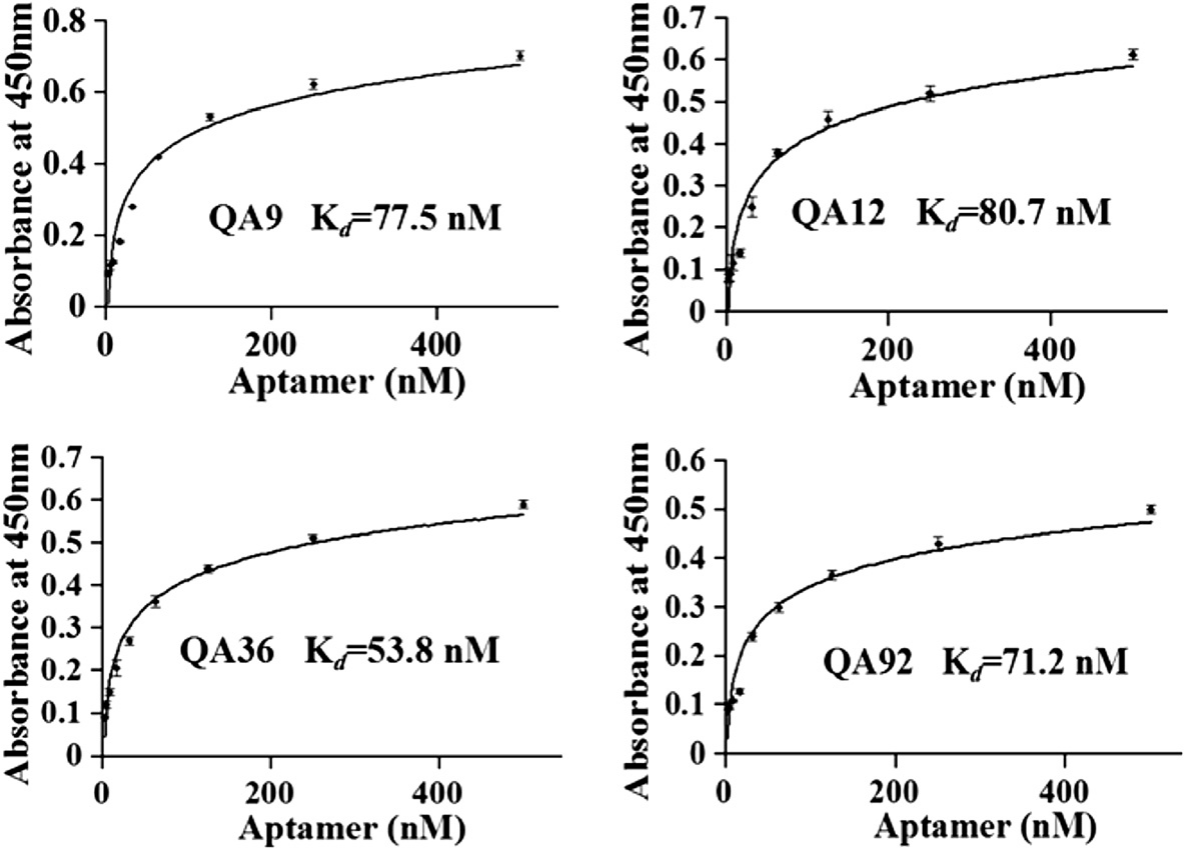
Affinity of STIV-selected ssDNA aptamer interactions by ELISA. STIV was incubated with increasing concentrations of 5′-biotinylated aptamer. After addition of streptavidin-HRP, the amount of STIV-aptamer complex was calculated and graphed as a function of aptamer concentration. The graph was fit to the equation Y = Bmax*X/(*K*_*d*_ + X) using SigmaPlot software. Results for each aptamer was presented as the mean ± SD of three independent experiments

The binding affinities of the aptamers to STIV were assessed by ELISA using 5′-biotinylated aptamers. All the four selected aptamers showed high affinity for STIV, with calculated binding affinities (*K*_*d*_) of 77.5 nM for QA9, 80.7 nM for QA12, 53.8 nM for QA36, and 71.2 nM for QA92 (Fig. 3). Of the four aptamer candidates, the *K*_*d*_ value of QA36 indicated the highest affinity.

### Cytotoxic effects of aptamers *in vitro* and *in vivo*

**Fig. 4:**
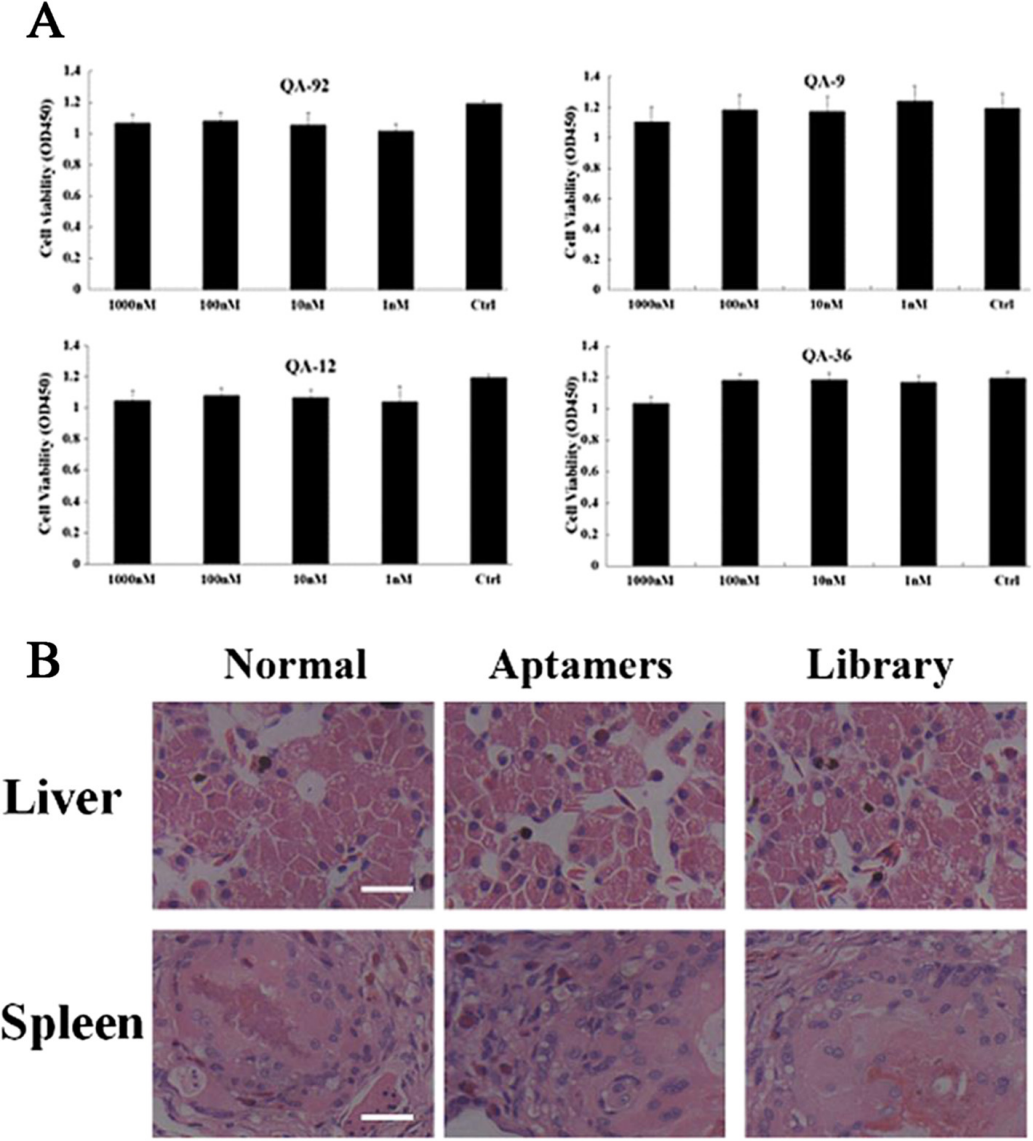
Aptamers exhibited no cytotoxic effects *in vitro* and *in vivo.* **a** DNA aptamers exhibited no cytotoxicity in cultured cells. The results for each group are presented as the mean ± SD of three independent experiments. **b** No pathological changes were found in liver and spleen tissues of aptamer-injected turtles, compared with normal control turtles. The scale bars indicate 20 μm

We incubated cells with the selected aptamers and then evaluated cell viability by the MTT method. There were no significant differences between the control and experimental groups, even at aptamer concentrations up to 1000 nM, suggesting that the aptamers were not cytotoxic (Fig. 4a). Furthermore, no turtles in the aptamer-treated or control groups had died 10 days p.i.. There were no pathological changes were in the liver or spleen in aptamer-injected turtles compared with controls, indicating that the selected aptamers had no cytotoxic effects *in vivo* (Fig. 4b).

### Inhibition of STIV infection by aptamers *in vitro* and *in vivo*

**Fig. 5:**
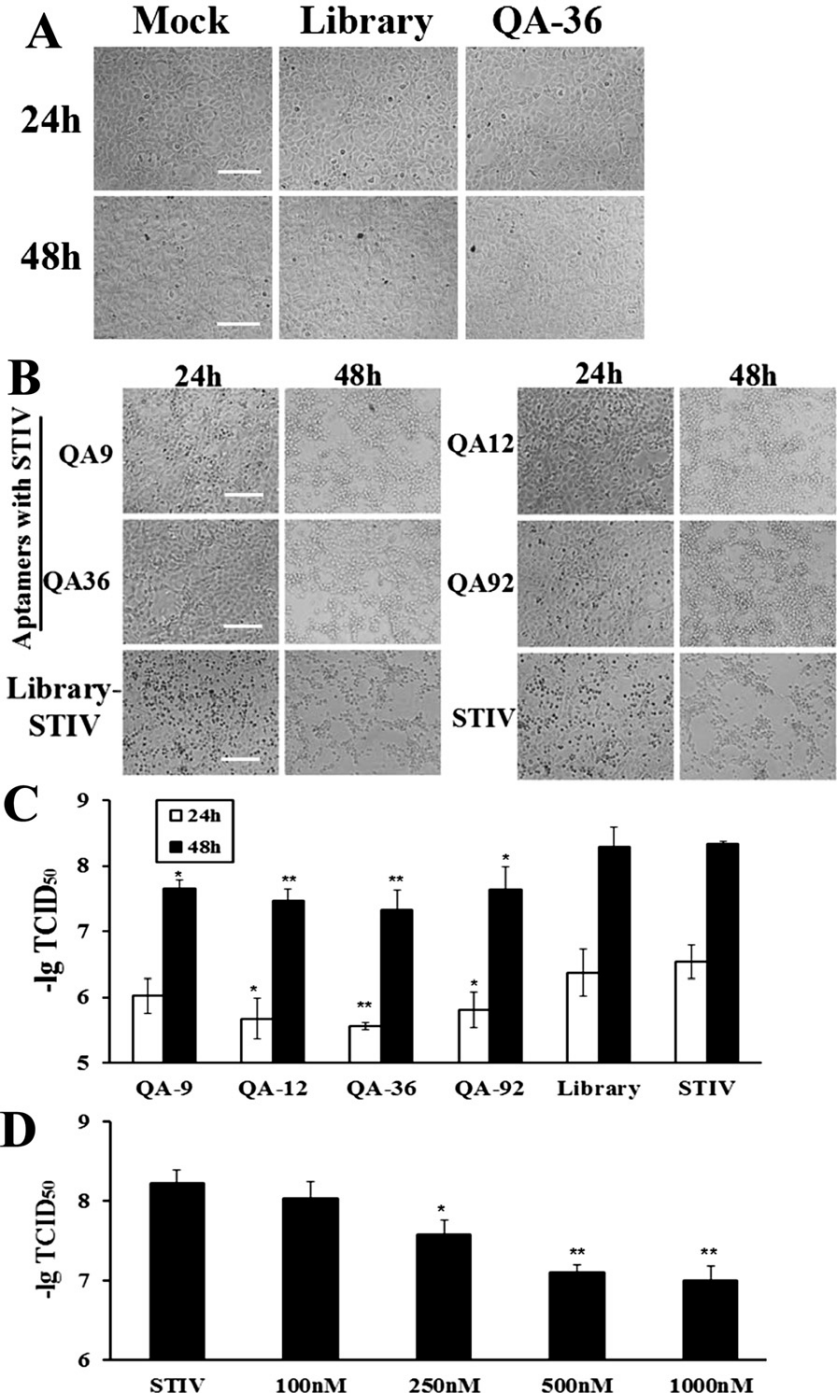
Selected aptamers inhibited STIV infection in cultured cells. **a** Morphology of FHM cells following treatment with the SELEX library or selected aptamers. **b** Cell morphology and CPE after treatment with STIV previously incubated with or without selected aptamers. **c** Aptamers reduced virus titers in cultured cells. **d** QA-36 aptamer inhibited STIV infection in a dose-dependent manner. The results for each group are presented as the mean ± SD of three independent experiments. (**P < 0.01; *P < 0.05; bars = 50 μm)

Cells without any treatment were served as mock group and grew normally. Cells incubated with SELEX library or aptamers also maintained normal growth, it indicated that selected aptamers exhibited no cytotoxic effects in cell cultures, which was consistent with the results of Fig. 4. (Fig. 5a). The inhibitory effects of aptamers was shown in Fig. 5b. There were significant CPEs in control cells incubated with STIV alone, when our aptamers were added, although there were some CPEs, the CPEs were much less than the control groups, which means STIV infection could be partially inhibited by the selected aptamers (Fig. 5b). Virus titers were significantly reduced by all the aptamers at 48 h post-infection, with QA-36 displaying the greatest inhibitory effect on STIV among the tested aptamers (Fig. 5c).

QA-36 was incubated with STIV at concentrations of 100, 250, 500, or 1000 nM and then added to the cells. The virus titers at 48 h p.i. showed that QA-36 inhibited STIV infection in a dose-dependent manner (Fig. 5d).

### Inhibition of STIV infection *in vivo* by QA-36

**Fig. 6:**
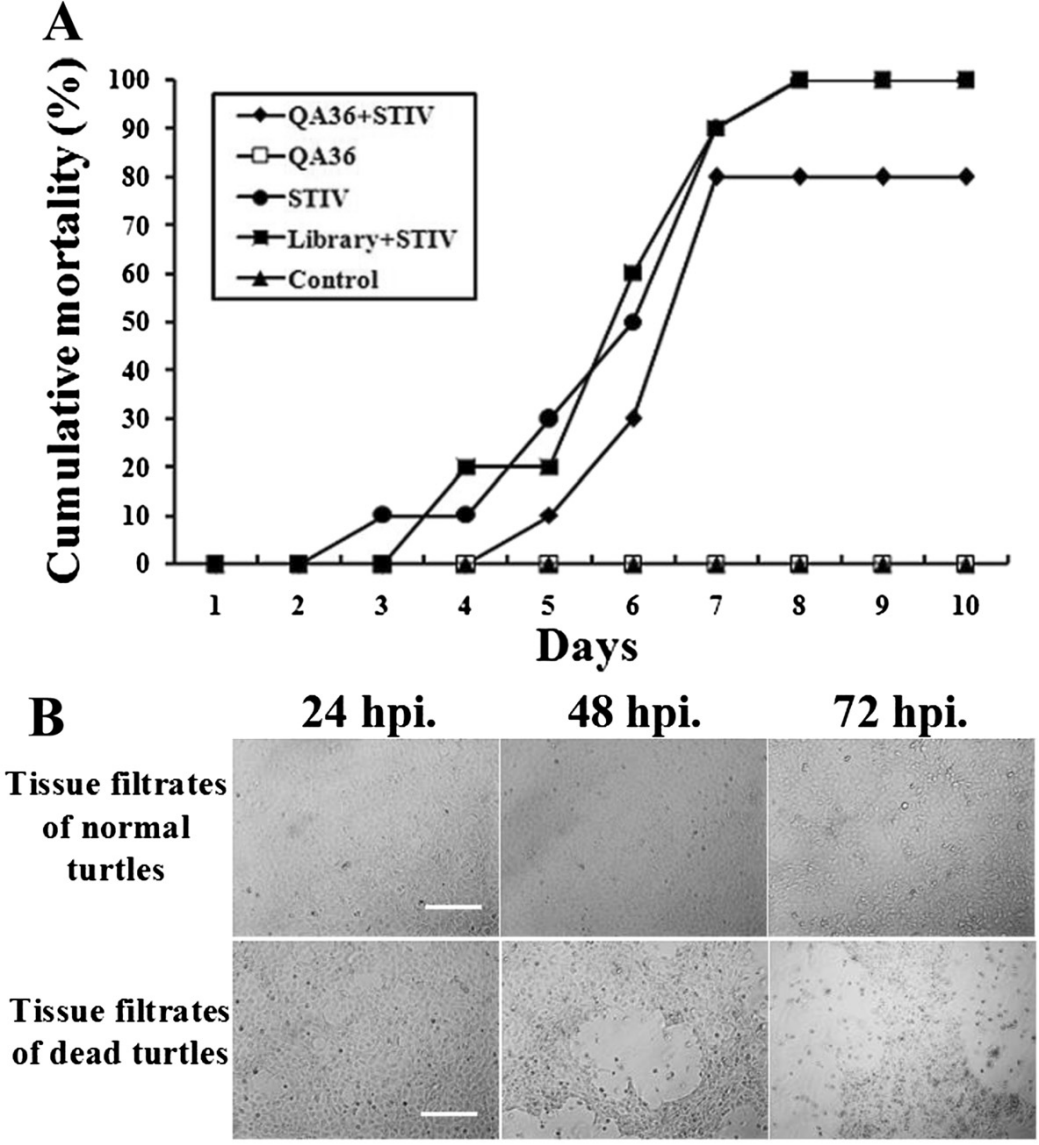
QA-36 inhibited STIV infection in cultured turtles. **a** Cumulative mortality of turtles was recorded daily up to 10 days p.i.. QA-36 slowed the rate of STIV infection and reduced the cumulative mortality by 20 %. **b** Filtrates collected from ground liver and spleen tissues of dead turtles exerted a CPE in FHM cells. (Bars = 100 μm)

QA-36 had the highest affinity for (Fig. 3) and demonstrated the most effective inhibition of STIV (Fig. 5d), and its inhibitory effects against STIV in soft-shelled turtles were therefore studied further. Turtles injected with STIV alone showed 10 % mortality on day 3, increasing to 100 % on day 8 p.i.. In contrast, no turtles injected with the mixtures of QA-36 and STIV died until day 5 p.i. and the cumulative mortality was 80 % (Fig. 6a), suggesting that QA-36 inhibited STIV infection *in vivo.*

Red congestion, bleeding, and ulceration as typical characteristics of STIV infection were evident in the neck, shell and mouth of the dead turtles, and dissection revealed red congestion and bleeding in the liver and spleen. Filtrates from the ground liver and spleen of dead turtles after 24 h p.i. demonstrated a CPE when incubated with FHM cells at 28 °C, and this effect was more significant after 48 h p.i. (Fig. 6b).

### STIV aptamer QA-12 specifically recognized STIV-infected cells and tissues

**Fig. 7:**
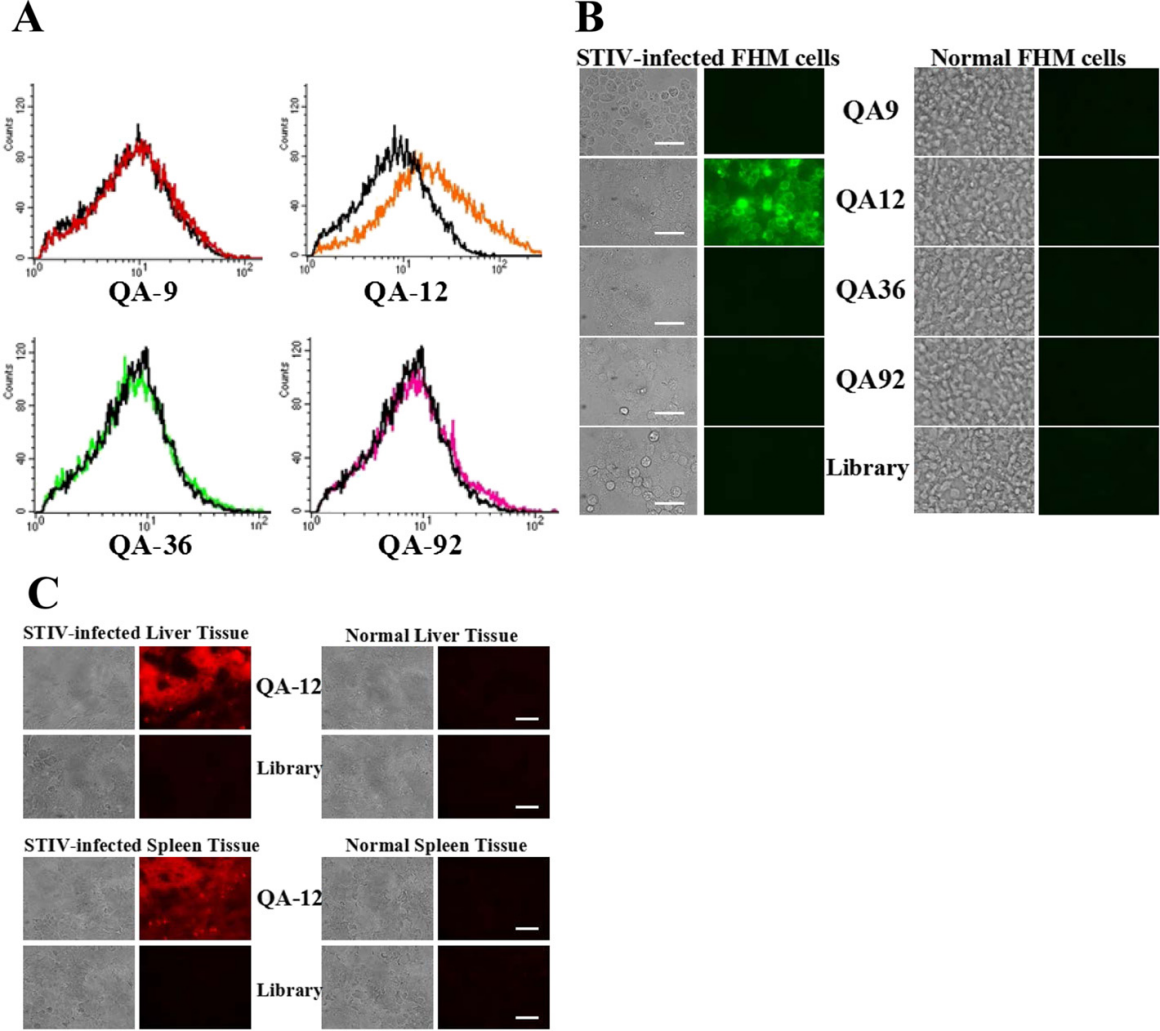
Some selected aptamers bound STIV-infected FHM cells with a high level of specificity. **a** Fluorescence intensities of four selected FITC-aptamers binding to infected FHM cells. The fluorescence intensity of QA-12 was increased in STIV-infected FHM cells compared with QA-9, QA-36 and QA-92. The FITC-labeled initial library incubated with STIV-infected FHM cells were used as the control (black). **b** Fluorescent images of four FITC-aptamers with STIV-infected FHM cells. QA-12 bound to STIV-infected cells but not to normal cells. QA-9, QA-36, QA-92 and the library bound to neither STIV-infected cells nor normal cells. The initial library incubated with infected cells and aptamers incubated with noamal cells were used as the negative controls. **c** Fluorescent images of four FITC-aptamers with STIV-infected turtle tissues. QA-12 bound to STIV-infected liver and spleen tissues but not to normal tissues. QA-9, QA-36 and QA-92 bound to neither STIV-infected tissues nor normal tissues. The initial library was used as a negative control. Left: bright field; right: fluorescent. (Bars = 20 μm)

To determine if aptamers against STIV recognized STIV-infected cells, we incubated FITC-aptamers (300 nM) with STIV-infected FHM cells and monitored the process by flow cytometry. The FITC-labeled initial library incubated with STIV-infected FHM cells were used as the control. Compared to the control, the fluorescence intensity of QA-12 increased in STIV-infected FHM cells, though the effects of QA-9, QA-36 and QA-92 were less noticeable, which means only QA-12 bound to STIV-infected cells, while QA-9, QA-36, QA-92 and the library could not bind to STIV-infected cells (Fig. 7a). These results were verified by imaging infected cell-aptamer interactions (Fig. 7b). Aptamer QA-12 evolved against STIV could thus recognize STIV-infected FHM cells. Fluorescent images of liver and spleen tissues indicated that the selected aptamers bound STIV-infected liver and spleen tissues with high levels of specificity (Fig. 7c).

## Discussion

STIV is an important causative agent isolated from soft-shelled turtles (*T. sinensis*) with ‘red neck disease’ [1]. However, no commercial vaccine against STIV is currently available, and effective strategies for detecting and inhibiting STIV infection are thus urgently needed. Highly sensitive and specific aptamers have been shown to have many applications [36]. They have been developed as promising candidates for antiviral therapeutics, and have demonstrated impressive results in animal-based studies and clinical trials [8, 9, 11, 36].

In the present study, we generated four ssDNA aptamers targeting STIV. Compared with the purified-protein-based SELEX method, aptamers selected through whole-virus-based SELEX could bind unknown targets on the virus surface, and may thus serve as effective biomarkers for studying the STIV infectious mechanism. In addition, viral surface proteins retain their native conformations, which are critical for biological functions [28]. FITC-aptamers could successfully recognize and label STIV virus particles, indicating their potential as commercially available probes for the early and rapid diagnosis of viral infection. Moreover, aptamers may also provide an alternative method for the real-time imaging of virus infection pathways. To the best of our knowledge, this study provides the first report of the ability of aptamers to inhibit and recognize STIV infection.

Aptamers have complex three dimensional structures including hairpins, stem-loops, and bulges, and are maintained by hydrogen bonding, electrostatic interactions and hydrophobic forces, which form the basis for tight aptamer binding to their targets [6]. Through competing for targets, aptamers may affect other interactions, such as virus-cell attachment. Hwang et al. (2012) reported antiviral aptamers that may bind to virus glycoproteins responsible for mediating virus entry into the cell [20]. The aptamers selected in the current study formed representative stem-loops in their complex secondary structures. QA-36 had the lowest Δ*G* value, indicating that its secondary structure was the most stable. Regarding the inhibitory capacity of aptamers, we speculated that their stem-loops might form target-binding sites allowing them to bind to their targets and interfere with STIV infection.

Flow cytometry and fluorescent imaging confirmed that the aptamer QA-12 was able to recognize STIV-infected cells. We speculate that STIV changes and rebuilds cell structures through the process of infection, causing some viral proteins or cell surface antigens to appear on the membranes of infected cells, which subsequently become part of the STIV envelope during its release. Aptamers bound to targets on both the infected-cell membrane, and the STIV envelope during the selection process. Aptamers are thus suitable molecular probes for the development of highly-specific diagnostics for detecting STIV infections.

The key to developing therapeutic reagents for *in vivo* application lies in their high specificity, low immunogenicity and lack of toxicity, thus reducing the side-effects associated with non-specific drug targeting. As highly-specific probes, aptamers are promising candidates for the targeted delivery of therapeutic reagents to the pathogen [37–39]. In this study, we confirmed that the selected aptamers had no cytotoxic effects either *in vitro* or *in vivo*, consistent with previous reports [22, 34]. Furthermore, QA-12 could recognize STIV-infected cells, and the use of aptamers in delivery vehicles provides a means of conferring selectivity to cell-type-specific interactions. This could also result in cellular internalization of the aptamers by the target cells, thereby increasing the therapeutic efficacy and reducing potential toxicities of the delivered drug. The molecular characteristics of cells infected by viruses, especially at the proteomic level, are critical for our understanding viral pathogenesis and for designing targeted therapies [16–19]. Further studies are therefore warranted to identify the viral mechanisms disrupted by binding of the aptamer QA-12.

## Conclusions

We generated DNA aptamers that bound STIV with a high level of specificity, providing an alternative means for identifying STIV and for real-time imaging of the virus-infection pathways. The selected aptamers inhibited STIV infection *in vitro* and *in vivo*, withQA-36 representing a particularly promising candidate therapeutic agents for blocking STIV infection. QA-12 also recognized STIV-infected cells and tissues, and could therefore be developed as candidate agents for investigating STIV pathogenesis, drug development, and medical therapies for STIV infection. This study provides the first evidence for aptamers with the capacity to inhibit and recognize STIV infection.

## Abbreviations

STIV: Soft-shelled turtle iridovirus
SELEX: Systematic evolution of ligands by exponential enrichment
*K*_*d*_: The apparent equilibrium dissociation constants
SGIV: Singapore grouper iridovirus
FHM: Fathead minnow cells
TCID_50_: 50 % Tissue culture infective dose
N50: Randomized 50-nucleotide sequence
FITC: Fluorescein isothiocyanate
TAMRA: Tetramethyl-6-carboxyrhodamine
PVDF: Polyvinylidene difluoride filter
PCR: Polymerase chain reaction
EMSA: Electrophoretic mobility shift assay
ELISA: Enzyme-linked immunosorbent assay
HRP: Horseradish peroxidase
MOI: Multiplicity of infection
p.i.: Post-infection
CPE: Cytopathic effect
PBS: Phosphate-buffered saline
SD: Standard deviation
Δ*G*: Free energy

## References

[1] Chen ZY, Zheng JC, Jiang YL (1999). A new iridovirus isolated from soft-shelled turtle. Virus Res.

[2] Du WG, Zhao B, Chen Y, Shine R (2011). Behavioral thermoregulation by turtle embryos. Proc Natl Acad Sci U S A.

[3] Fritz U, Gong S, Auer M, Kuchling G, Schneeweiß N, Hundsdörfer AK (2010). The world’s economically most important chelonians represent a diverse species complex (Testudines: Trionychidae: Pelodiscus). Org Divers Evol.

[4] Magadán-Mompó S, Sánchez-Espinel C, Gambón-Deza F (2013). Immunoglobulin genes of the turtles. Immunogenetics.

[5] Nagashima H, Sugahara F, Takechi M, Ericsson R, Kawashima-Ohya Y, Narita Y (2009). Evolution of the turtle body plan by the folding and creation of new muscle connections. Science.

[6] Ellington AD, Szostak JW (1990). *In vitro* selection of RNA molecules that bind specific ligands. Nature.

[7] Syed MA, Pervaiz S (2010). Advances in Aptamers. Oligonucleotides.

[8] Zhou J, Rossi JJ (2011). Cell-specific aptamer-mediated targeted drug delivery. Oligonucleotides.

[9] Zhou J, Rossi JJ (2012). Therapeutic potential of aptamer-siRNA conjugates for treatment of HIV-1. BioDrugs.

[10] Balogh Z, Lautner G, Bardoczy V, Komorowska B, Gyurcsanyi RE, Meszaros T (2010). Selection and versatile application of virus-specific aptamers. FASEB J.

[11] Bunka DHJ, Platonova O, Stockley PG (2010). Development of aptamer therapeutics. Curr Opin Pharmacol.

[12] Chou SH, Chin KH, Wang AH (2005). DNA aptamers as potential anti-HIV agents. Trends Biochem Sci.

[13] Liang Y, Zhang ZP, Wei HP, Hu QX, Deng JY, Guo DY (2011). Aptamer beacons for visualization of endogenous protein HIV-1 reverse transcriptase in living cells. Biosens Bioelectron.

[14] Xiao ZY, Farokhzad OC (2012). Aptamer-Functionalized Nanoparticles for Medical Applications: Challenges and Opportunities. ACS Nano.

[15] Abósa B, Castroa R, González Granja A, Havixbeckb JJ, Barredab DR, Tafalla C (2015). Early Activation of Teleost B Cells in Response to Rhabdovirus Infection. J Virol.

[16] Gerold G, Pietschmann T (2014). The HCV life cycle: *in vitro* tissue culture systems and therapeutic targets. Dig Dis.

[17] Karst SM, Zhu S, Goodfellow IG (2015). The molecular pathology of noroviruses. J Pathol.

[18] Seeger C, Mason WS (2015). Molecular biology of hepatitis B virus infection. Virology.

[19] Verdaguer N, Ferrero D, Murthy MR (2014). Viruses and viral proteins. IUCrJ.

[20] Hwang SD, Midorikawa N, Punnarak P, Kikuchi Y, Kondo H, Hirono I (2012). Inhibition of hirame rhabdovirus growth by RNA aptamers. J Fish Dis.

[21] Porntep P, Mudjekeewis DS, Seong DH, Hidehiro K, Ikuo H, Yo K (2012). RNA aptamers inhibit the growth of the fish pathogen viral hemorrhagic septicemia virus (VHSV). Mar Biotechnol.

[22] Li PF, Yan Y, Wei SN, Wei J, Gao R, Huang X (2014). Isolation and characterization of a new class of DNA aptamers specific binding to Singapore grouper iridovirus (SGIV) with antiviral activities. Virus Res.

[23] Huang YH, Huang XH, Liu H, Gong J, Ouyang ZL, Cui HC (2009). Complete sequence determination of a novel reptile iridovirus isolated from soft-shelled turtle and evolutionary analysis of Iridoviridae. BMC Genomics.

[24] Qin QW, Chang SF, Ngoh-Lim GH, Gibson-Kueh S, Shi C, Lam TJ (2003). Characterization of a novel ranavirus isolated from grouper Epinephelus tauvina. Dis Aquat Organ.

[25] Huang XH, Huang YH, Ouyang ZL, Qin QW (2011). Establishment of a cell line from the brain of grouper (Epinephelus akaara) for cytotoxicity testing and virus pathogenesis. Aquaculture.

[26] Qin QW, Wu TH, Jia TL, Hegde A, Zhang RQ (2006). Development and characterization of a new tropical marine fish cell line from grouper, Epinephelus coioides susceptible to iridovirus and nodavirus. J Virol Methods.

[27] Reed LJ, Muench H (1938). A simple method of estimating fifty percent endpoints. Am J Epidemiol.

[28] Pan W, Craven RC, Qiu Q, Wilson CB, Wills JW, Golovine S (1995). Isolation of virus-neutralizing RNAs from a large pool of random sequences. Proc Natl Acad Sci U S A.

[29] Shangguan DH, Li Y, Tang Z, Cao ZC, Chen HW, Mallikaratchy P (2006). Aptamers evolved from live cells as effective molecular probes for cancer study. Proc Natl Acad Sci U S A.

[30] Cui ZQ, Ren Q, Wei HP, Chen Z, Deng JY, Zhang ZP (2011). Quantum dot-aptamer nanoprobes for recognizing and labeling influenza A virus particles. Nanoscale.

[31] Park JH, Jee MH, Kwon OS, Keum SJ, Jang SK (2013). Infectivity of hepatitis C virus correlates with the amount of envelope protein E2: development of a new aptamer-based assay system suitable for measuring the infectious titer of HCV. Virology.

[32] Toscano-Garibay JD, Benítez-Hess ML, Alvarez-Salas LM (2011). Isolation and characterization of an RNA aptamer for the HPV-16 E7 oncoprotein. Arch Med Res.

[33] Liang HR, Liu Q, Zheng XX (2013). Aptamers targeting rabies virus-infected cells inhibit viral replication both *in vitro* and *in vivo*. Virus Res.

[34] Liang HR, Hu GQ, Zhang T, Yang YJ, Zhao LL, Qi YL (2012). Isolation of ssDNA aptamers that inhibit rabies virus. Int Immunopharmacol.

[35] Shangguan DH, Meng L, Cao ZC, Xiao Z, Fang X, Li Y (2008). Identification of liver cancer-specific aptamers using whole live cells. Anal Chem.

[36] Bunka DHJ, Stockley PG (2006). Aptamers come of age-at last. Nature Rev Microbiol.

[37] Chu TC, Twu KY, Ellington AD, Levy M (2006). Aptamer mediated siRNA delivery. Nucleic Acids Res.

[38] Xiao ZY, Shangguan DH, Cao ZH, Fang XH, Tan WH (2008). Cell-Specific Internalization Study of an Aptamer from Whole Cell Selection. Chem Eur J.

[39] Zhang KJ, Sefah K, Tang LL, Zhao ZL, Zhu GZ, Ye M (2012). A Novel Aptamer Developed for Breast Cancer Cell Internalization. ChemMedChem.

